# Arrhythmia and cardiomyopathy risk in Taiwan with complementary biobank evidence on thyroid genetic susceptibility: an integrative population-based framework

**DOI:** 10.1530/EC-26-0366

**Published:** 2026-08-17

**Authors:** Yi-Chang Lin, Yao-Ching Huang, Ya-Ting Yang, Su-Wen Chuang, Tsu-Hsuan Weng, Chun-Teng Tsai, Chi-Hsiang Chung, Chang-Huei Tsao, Wu-Chien Chien, Chien-Sung Tsai

**Affiliations:** ^1^ Division of Cardiovascular Surgery, Department of Surgery, Tri-Service General Hospital, National Defense Medical University, Taipei, Taiwan; ^2^ Division of Transplantation Surgery, Department of Transplantation, Tri-Service General Hospital, National Defense Medical University, Taipei, Taiwan; ^3^ Department of Medical Research, Tri-Service General Hospital, National Defense Medical University, Taipei, Taiwan; ^4^ School of Public Health, College of Public Health, National Defense Medical University, Taipei, Taiwan; ^5^ Department of Chemical Engineering and Biotechnology, National Taipei University of Technology (Taipei Tech), Taipei, Taiwan; ^6^ College of Public Health, National Defense Medical University, Taipei, Taiwan; ^7^ Graduate Institute of Life Sciences, College of Biomedical Sciences, National Defense Medical University, Taipei, Taiwan; ^8^ Department and Graduate Institute of Microbiology & Immunology, College of Biomedical Sciences, National Defense Medical University, Taipei, Taiwan; ^9^ Graduate Institute of Medicine, National Defense Medical University, Taipei, Taiwan; ^10^ Taiwanese Injury Prevention and Safety Promotion Association (TIPSPA) Taipei, Taiwan; ^11^ Graduate Institute of Public Health, College of Public Health, National Defense Medical University, Taipei, Taiwan; ^12^ Department of Public Health, College of Medicine, Fu-Jen Catholic University, New Taipei City, Taiwan

**Keywords:** arrhythmia, cardiomyopathy, thyroid dysfunction, polygenic risk score (PRS), endocrine genetics, precision medicine

## Abstract

**Background:**

Arrhythmia-induced cardiomyopathy (AiCM) is a potentially reversible cause of ventricular dysfunction; however, only a subset of patients with arrhythmia develop cardiomyopathy. Emerging evidence suggests that endocrine factors, particularly thyroid dysfunction with genetic susceptibility, may contribute to inter-individual variability in arrhythmia-related myocardial outcomes.

**Methods:**

We performed a dual-cohort population-based study using the National Health Insurance Research Database (NHIRD, 2000–2015) and the Taiwan Biobank (TWB). In NHIRD, we examined the association between newly diagnosed arrhythmia and incident cardiomyopathy using Cox proportional hazards models. In TWB, genome-wide data, thyroid-stimulating hormone (TSH), polygenic risk scores (PRSs), lifestyle factors, and metabolic comorbidities were analyzed using multivariable regression and interaction models to assess determinants of thyroid dysfunction.

**Results:**

In the NHIRD cohort, arrhythmia was associated with a significantly increased risk of incident cardiomyopathy (adjusted hazard ratio (aHR): 2.49, 95% CI: 1.94–2.96), with atrial fibrillation showing the strongest association among arrhythmia subtypes. In the TWB cohort, a higher thyroid polygenic risk score was strongly associated with thyroid dysfunction (adjusted odds ratio (aOR): 6.64, 95% CI: 5.86–7.52). The association between genetic susceptibility and thyroid dysfunction was further modified by metabolic and lifestyle factors, including diabetes, hyperlipidemia, and dietary patterns. Genome-wide analysis identified multiple loci associated with thyroid-stimulating hormone regulation, consistent with a polygenic architecture of thyroid endocrine traits.

**Conclusion:**

Arrhythmia was associated with an increased risk of cardiomyopathy in a nationwide cohort, while thyroid genetic susceptibility was strongly associated with thyroid dysfunction in a biobank cohort and modified by metabolic and lifestyle factors. These findings provide complementary population-level evidence of parallel cardiovascular and endocrine–genetic associations. Because the two cohorts were not individually linked, causal inference cannot be established. The results support a systems-level framework of endocrine–cardiac interaction and suggest that integrated clinical and genetic risk assessment may help identify individuals who warrant closer monitoring.

## Introduction

Cardiomyopathy is a heterogeneous myocardial disorder that can progress to heart failure, malignant arrhythmia, and sudden cardiac death, representing a major cause of cardiovascular morbidity and mortality worldwide (1, 2, 3). Among potentially reversible forms, arrhythmia-induced cardiomyopathy (AiCM) has been increasingly recognized as a distinct clinical entity, in which sustained or frequent arrhythmias – including atrial fibrillation (AF), atrial flutter, supraventricular tachycardia, and ventricular ectopy – lead to left ventricular dysfunction (1, 2, 4). Mechanistically, AiCM is not solely driven by tachycardia, but also involves irregular electrical activation, calcium-handling dysregulation, neurohormonal activation, and fibro-inflammatory myocardial remodeling (1, 2, 4, 5).

Thyroid hormones play a central role in cardiovascular homeostasis by modulating cardiac electrophysiology, autonomic tone, myocardial contractility, and vascular resistance. Both overt thyroid dysfunction and subclinical thyroid dysfunction have been associated with an increased arrhythmogenic risk, particularly atrial fibrillation, highlighting a strong endocrine–electrophysiological interaction (5, 6, 7). Hyperthyroidism is a well-established reversible cause of AF, while subclinical thyroid dysfunction has also been linked to an increased cardiovascular risk, supporting a graded relationship between thyroid status and cardiac rhythm disorders (6, 7, 8, 9).

Genetic susceptibility contributes significantly to inter-individual variability in thyroid function and arrhythmia risk. Genome-wide association studies (GWASs) have identified multiple loci influencing thyroid-stimulating hormone (TSH) regulation and thyroid dysfunction, while polygenic risk scores (PRSs) have been developed to quantify inherited susceptibility to endocrine traits (10, 11). These genetic factors may act upstream to influence thyroid function, which in turn may modulate susceptibility to arrhythmia and potentially contribute to arrhythmia-induced myocardial remodeling.

Despite these mechanistic insights, the integrated relationship among thyroid genetic susceptibility, endocrine dysfunction, arrhythmia, and cardiomyopathy remains incompletely understood at the population level. Previous studies have largely examined these domains separately, focusing either on arrhythmia–cardiomyopathy associations or on thyroid–arrhythmia relationships, without integrating genetic and endocrine dimensions within a unified framework.

To address this gap, we leveraged two large Taiwanese population-based resources: the National Health Insurance Research Database (NHIRD), which provides longitudinal cardiovascular outcome data, and the Taiwan Biobank (TWB), which contains genome-wide genetic, endocrine, lifestyle, and metabolic information (11, 12). This dual-cohort design allows complementary examination of cardiovascular and endocrine–genetic risk architectures in independent populations.

Because individual-level linkage between NHIRD and TWB was not available, this study does not directly infer causal pathways. Instead, we propose a hypothesis-generating, systems-level framework, in which thyroid genetic susceptibility and endocrine dysfunction may represent upstream modifiers of arrhythmogenic vulnerability and myocardial remodeling risk. This integrative approach enables exploration of parallel biological associations across endocrine and cardiovascular systems while minimizing shared-data bias (12, 13).

## Materials and methods

### Study design and data sources

We conducted an integrative population-based study using two independent Taiwanese resources: the NHIRD and the TWB. Analyses were performed separately due to lack of individual-level linkage and synthesized using a systems-level interpretative framework (11, 12, 13). A schematic overview is provided in Supplementary Fig. S1 (see section on Supplementary materials given at the end of the article).

### NHIRD cohort

Adults aged ≥20 years with newly diagnosed arrhythmia (2001–2014) were identified using ICD-9-CM codes for atrial fibrillation, atrial flutter, supraventricular tachycardia, ventricular tachycardia, and ventricular fibrillation. Propensity score matching (1:4) was applied (14, 15, 16).

### Outcome definition

Incident cardiomyopathy was defined using ICD-9-CM 425.xx in ≥1 inpatient or ≥2 outpatient claims (16, 17).

### TWB cohort

Participants with genome-wide genotyping, TSH measurement, and lifestyle data were included (*n* = 17,571). Thyroid dysfunction was defined as TSH 0.4–4.0 mIU/L range violation (18, 19).

### GWASs and PRS

GWASs used linear regression adjusted for covariates, significance threshold *P* < 5 × 10^−8^ (20). PRS was constructed using external GWAS weights (21, 22, 23).

### Lifestyle and metabolic variables

Smoking, alcohol, diet, physical activity, and comorbidities were assessed via standardized questionnaires (24, 25, 26).

### Statistical analysis

Cox regression estimated aHR in NHIRD; logistic regression estimated aOR in TWB. Kaplan–Meier with log-rank test evaluated survival. Analyses used SAS, PLINK, and R.

### Ethics

This study was approved by IRB; NHIRD consent was waived because data were encrypted and de-identified; and TWB participants provided written informed consent (27).

## Results

### Cohort characteristics and survival

The NHIRD cohort included 333,080 individuals (66,616 with arrhythmia and 266,464 matched controls). During a mean follow-up of 7.9 years, 3,873 participants developed cardiomyopathy.

**Figure 1:**
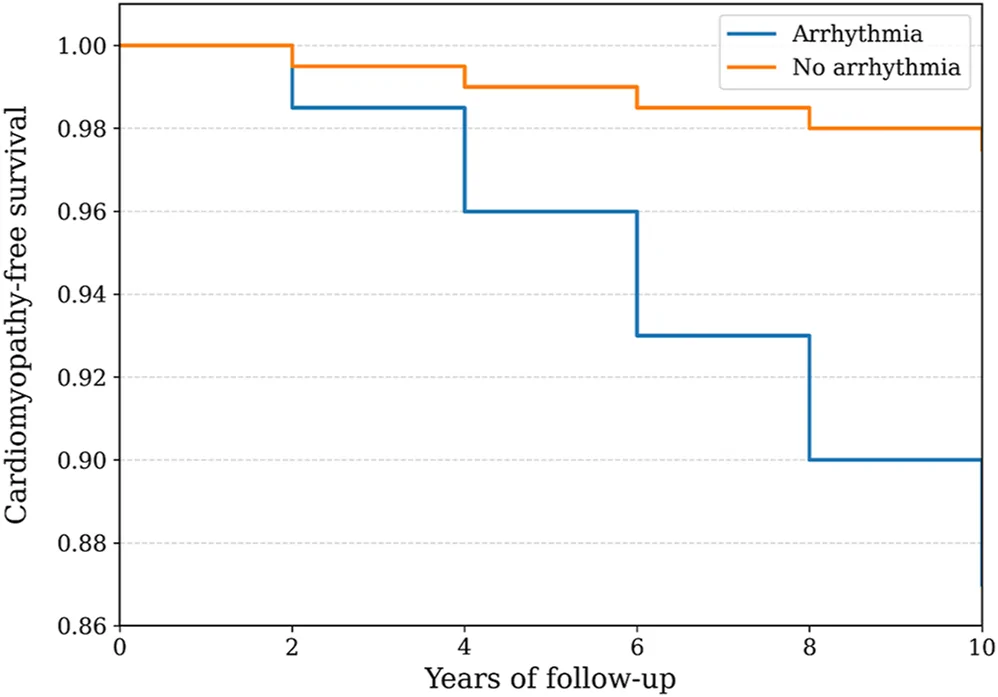
Cardiomyopathy-free survival in participants with and without arrhythmia.

Kaplan–Meier analysis demonstrated significantly reduced cardiomyopathy-free survival in the arrhythmia group compared with controls (log-rank *P* < 0.001) (Fig. 1). Early divergence between groups emerged and persisted throughout follow-up, indicating a sustained association over time.

### Association between arrhythmia and cardiomyopathy

Arrhythmia was independently associated with an increased risk of incident cardiomyopathy (aHR: 2.49, 95% CI: 1.94–2.96; *P* < 0.001) (Fig. 1, Table 1).

**Table 1:** Stratified association between arrhythmia and incident cardiomyopathy.

Subgroup	Events in arrhythmia group	Events in control group	aHR	95% CI	*P*-value
Overall	2,569	1,304	2.49	1.94–2.96	<0.001
Male	1,589	855	2.34	1.83–2.79	<0.001
Female	980	449	2.75	2.15–3.29	<0.001
Age 20–49 years	372	210	2.18	1.69–2.62	<0.001
Age 50–64 years	1,068	569	2.37	1.82–2.80	<0.001
Age ≥65 years	1,129	525	2.79	2.15–3.29	<0.001

Note: Adjusted hazard ratios were estimated using Cox proportional hazards regression models. The control group without arrhythmia served as the reference group in each stratum.

aHR, adjusted hazard ratio; CI, confidence interval.

These associations were consistent across sex and age strata, with higher effect estimates observed in older individuals. Detailed subgroup estimates are presented in Table 1.

### Arrhythmia subtypes and risk

**Figure 2:**
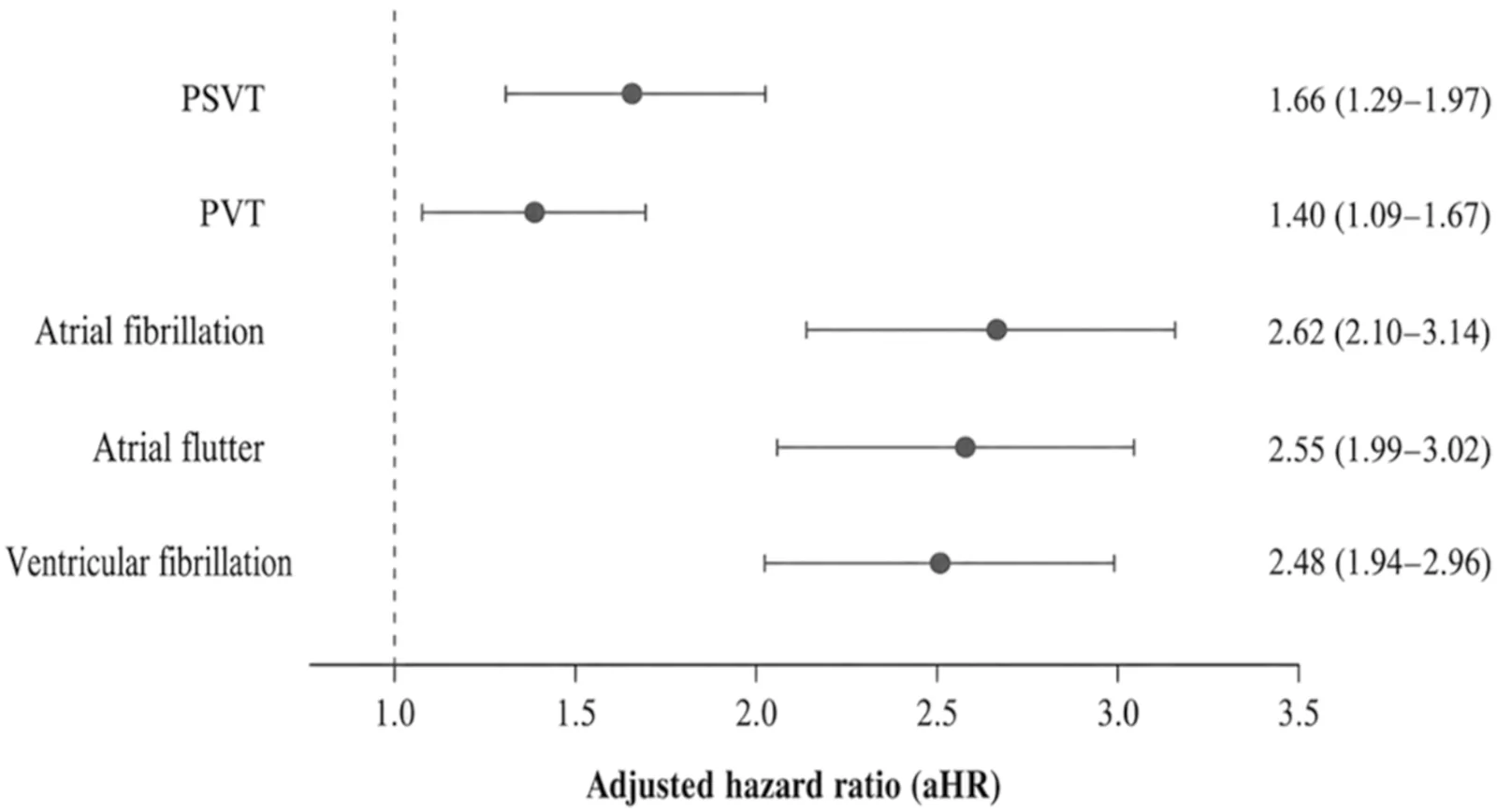
Risk of incident cardiomyopathy according to arrhythmia subtype.

Risk of cardiomyopathy varied across arrhythmia subtypes. Atrial fibrillation showed the strongest association, followed by atrial flutter and ventricular fibrillation. Supraventricular and paroxysmal tachycardias were also significantly associated, although with lower effect sizes (Fig. 2).

Overall, sustained atrial arrhythmias demonstrated stronger associations with cardiomyopathy compared with transient tachyarrhythmias.

### Genome-wide association of thyroid function

**Figure 3:**
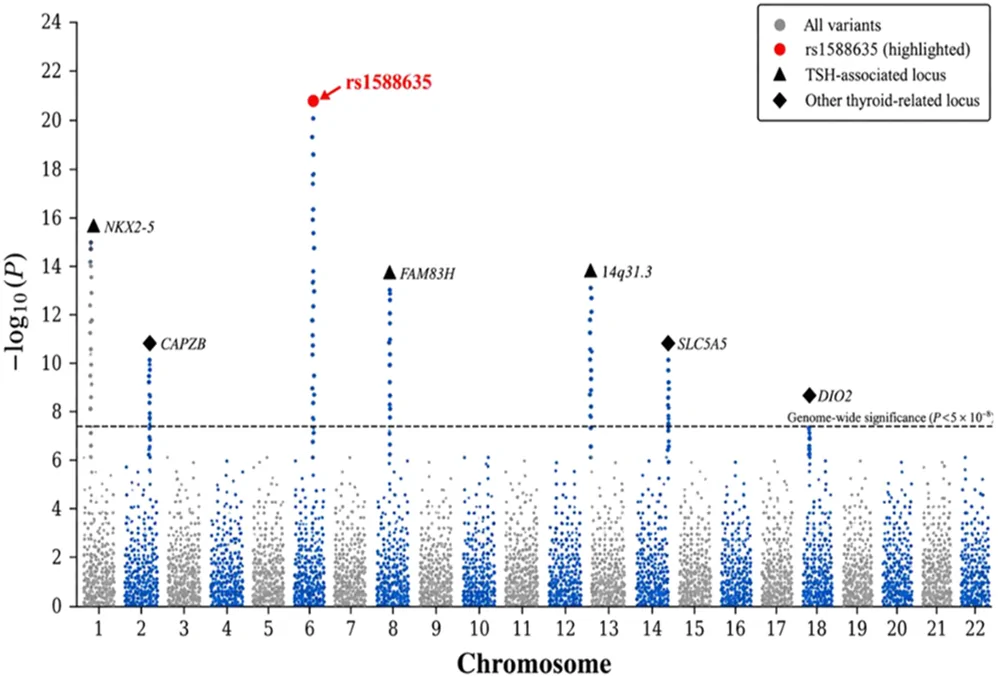
Genome-wide association analysis of serum thyrotropin with labeled thyroid-related loci.

Multiple genome-wide significant loci (*P* < 5 × 10^−8^) were identified for TSH regulation, including PTCSC2 (FOXE1 locus), TSHR, PDE8B, PDE10A, VAV3, and DIO2. These findings support a polygenic architecture underlying thyroid endocrine regulation (Fig. 3, Supplementary Table S1).

A comprehensive list of significant loci and annotations is provided in Supplementary Table S1.

### Polygenic risk score and thyroid dysfunction

**Figure 4:**
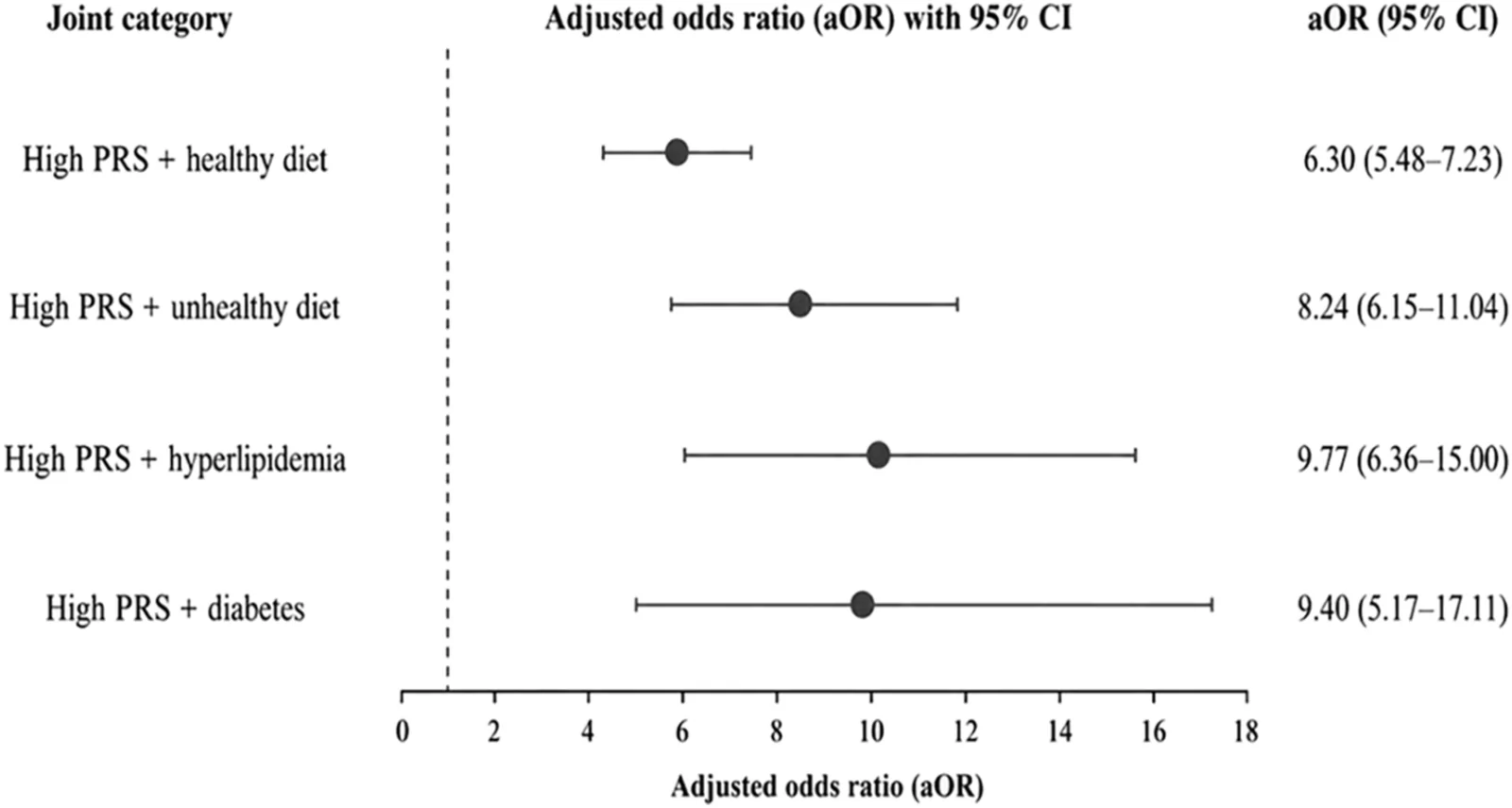
Joint effects of thyroid polygenic risk score and lifestyle/metabolic factors on thyroid dysfunction.

A higher thyroid PRS was strongly associated with thyroid dysfunction (aOR: 6.64, 95% CI: 5.86–7.52), indicating a substantial contribution of inherited susceptibility to endocrine disease risk (Fig. 4).

### Gene–lifestyle–metabolic interaction

Significant interaction effects were observed between thyroid PRS and lifestyle/metabolic factors. Individuals with a high PRS combined with diabetes, hyperlipidemia, or unhealthy dietary patterns exhibited the highest risk of thyroid dysfunction (Fig. 4).

In contrast, healthier lifestyle patterns were associated with attenuation of genetic risk, suggesting partial environmental modulation of inherited susceptibility.

### Integrated framework

**Figure 5:**
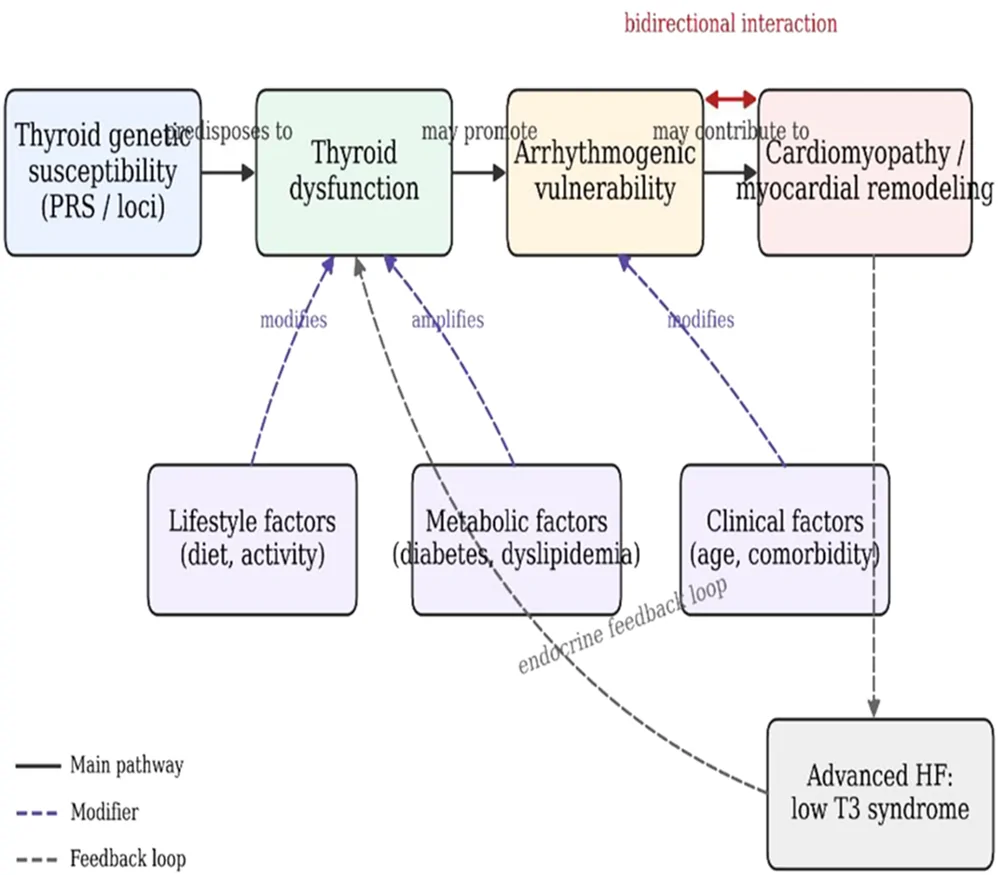
Hypothesis-generating endocrine–genetic–arrhythmia–cardiomyopathy framework with bidirectional interactions.

These findings support a biologically plausible parallel continuum linking thyroid genetic susceptibility, thyroid dysfunction, arrhythmia, and cardiomyopathy (Fig. 5).

Because the datasets were not linked at the individual level, the observed associations should be interpreted as complementary rather than causal.

## Discussion

This integrative study provides complementary population-level evidence linking arrhythmia-associated cardiomyopathy risk with thyroid genetic susceptibility and endocrine–metabolic modulation. Using two independent Taiwanese cohorts, we observed that arrhythmia was associated with an increased risk of cardiomyopathy in the NHIRD cohort, while thyroid polygenic susceptibility was strongly associated with thyroid dysfunction and modified by metabolic and lifestyle factors in the Taiwan Biobank cohort. These parallel findings suggest shared biological susceptibility across endocrine and cardiovascular systems rather than a directly inferred causal pathway.

Because the NHIRD and TWB datasets were not individually linked, the results should be interpreted as complementary associations supporting a systems-level hypothesis of cardiometabolic–endocrine interaction (28, 29, 30).

### Arrhythmia and risk of cardiomyopathy

Our nationwide findings are consistent with prior evidence that AiCM represents a clinically important and potentially reversible cause of ventricular dysfunction (28, 29, 30). The observed associations across sex and age strata suggest that arrhythmia burden may be associated with cardiomyopathy risk at the population level, particularly in older individuals and those with atrial fibrillation.

Mechanistically, AiCM is increasingly understood as a multifactorial process involving electrical dyssynchrony, calcium-handling abnormalities, energetic impairment, and neurohormonal activation rather than tachycardia alone (28, 29, 31). These mechanisms support progressive myocardial remodeling in the setting of sustained rhythm disturbance.

### Thyroid dysfunction and arrhythmic susceptibility

Thyroid hormones exert broad effects on cardiac electrophysiology, autonomic regulation, and myocardial contractility. Epidemiological and meta-analytic studies have demonstrated associations between subclinical thyroid dysfunction and atrial fibrillation, and hyperthyroidism remains a well-established reversible cause of atrial arrhythmia (32, 33).

In the present study, TWB data demonstrated that inherited thyroid genetic susceptibility was strongly associated with thyroid dysfunction (34, 35). These findings extend prior evidence by suggesting that genetic architecture contributes to variability in endocrine status, which may indirectly relate to arrhythmic susceptibility through endocrine-mediated pathways.

Importantly, relationships among thyroid dysfunction, arrhythmia, and cardiomyopathy are likely bidirectional rather than strictly linear. Cardiac dysfunction may influence thyroid hormone metabolism, including low triiodothyronine states, while cardiomyopathy may increase susceptibility to recurrent arrhythmia. Accordingly, the conceptual framework in Fig. 5 should be interpreted as a dynamic interaction model rather than a deterministic causal pathway (28, 29, 30, 31, 32, 33).

### Genetic susceptibility, lifestyle, and metabolic interaction

Genome-wide analysis identified rs1588635 and related loci associated with TSH regulation, consistent with prior evidence supporting the polygenic architecture of thyroid function (34, 35). These variants reflect inherited susceptibility influencing endocrine regulation, which may indirectly relate to cardiovascular phenotypes through endocrine-mediated pathways.

Although individual-level linkage between genetic variants and arrhythmia outcomes was not available, inclusion of these loci in the polygenic risk score supports biological plausibility for shared endocrine–cardiac susceptibility (38, 39).

In addition, individuals with a high polygenic risk combined with adverse metabolic profiles, including diabetes, hyperlipidemia, and unhealthy dietary patterns, exhibited the highest risk of thyroid dysfunction. These findings are consistent with prior literature emphasizing the importance of modifiable metabolic factors in endocrine and cardiovascular disease susceptibility (18, 36, 37).

Clinically, PRS should not be interpreted deterministically. Instead, they may provide supplementary information for identifying individuals who could benefit from earlier endocrine monitoring and optimization of cardiometabolic risk factors, pending further validation (38, 39).

### Clinical implications

Integrated atrial fibrillation care increasingly emphasizes rhythm control, stroke prevention, and comorbidity management (40, 41). Our findings suggest that endocrine evaluation and metabolic risk optimization may be particularly relevant in patients with arrhythmia who present with early ventricular dysfunction or an increased cardiometabolic risk burden.

This interpretation aligns with evidence demonstrating that lifestyle and risk factor modification can reduce atrial fibrillation burden and improve clinical outcomes (31, 42, 43). However, the clinical application of genetic risk information remains investigational and requires further validation.

### Strengths and limitations

The primary strength of this study is the use of two large, independent Taiwanese population-based datasets, enabling complementary evaluation of cardiovascular and endocrine–genetic associations. The NHIRD provided longitudinal outcome data for arrhythmia and cardiomyopathy, while the Taiwan Biobank provided genetic, endocrine, and lifestyle information.

Several limitations should be acknowledged. First, the lack of individual-level linkage between NHIRD and TWB precluded mediation analyses and direct evaluation of causal pathways. Second, thyroid function in TWB was measured cross-sectionally, limiting temporal inference. Third, NHIRD diagnostic codes do not capture detailed clinical information, such as arrhythmia burden, imaging findings, or treatment strategies. Fourth, potential selection bias in biobank participation may limit generalizability. Finally, residual confounding cannot be excluded despite multivariable adjustment and propensity score matching.

### Future perspectives

Future studies should validate this framework in prospective cohorts integrating genomic, endocrine, electrophysiological, and imaging data. Such studies should include serial thyroid hormone measurements, continuous rhythm monitoring, echocardiographic assessment, and longitudinal follow-up for cardiomyopathy outcomes.

From a translational perspective, PRSs may eventually contribute to multimodal risk stratification when combined with clinical, metabolic, and lifestyle variables. However, clinical implementation requires external validation, evaluation of clinical utility, and careful consideration of ethical implications related to genetic risk disclosure.

## Conclusions

In this dual-cohort population-based study, arrhythmia was associated with an increased risk of incident cardiomyopathy in a nationwide Taiwanese cohort, while thyroid genetic susceptibility was strongly associated with thyroid dysfunction and modified by metabolic and lifestyle factors in an independent biobank cohort.

These findings provide complementary population-level evidence of parallel associations across cardiovascular and endocrine–genetic domains. Collectively, they support a biologically plausible parallel continuum linking thyroid genetic susceptibility, endocrine dysfunction, arrhythmia, and cardiomyopathy.

However, because the NHIRD and Taiwan Biobank cohorts were not individually linked, causal inference cannot be established. The findings should therefore be interpreted as hypothesis-generating evidence of shared biological susceptibility rather than a direct causal pathway.

Future studies integrating longitudinal genomic, endocrine, electrophysiological, and imaging data are needed to clarify temporal relationships and evaluate the clinical utility of combined endocrine–genetic–cardiac risk stratification.

## Supplementary materials





## Declaration of interest

The authors declare that there is no conflict of interest that could be perceived as prejudicing the impartiality of the work reported.

## Funding

This study was supported by the Tri-Service General Hospital Research Foundation (TSGH-A-115015; TSGH-B-115018); the sponsor had no role in the study.

## Author contribution statement

All authors contributed to study conception/design, data analysis or interpretation, manuscript drafting or critical revision, and approval of the final version.

## Ethical approval and consent to participate

The Tri-Service General Hospital Institutional Review Board approved this study (TSGHIRB No. E202616017). NHIRD consent was waived because data were encrypted and de-identified; TWB participants provided written consent.

## Availability of data and materials

Data are available from the corresponding author upon reasonable request and subject to database regulations.
