# Supplementary material for: Arrhythmia and cardiomyopathy risk in Taiwan with complementary biobank evidence on thyroid genetic susceptibility: an integrative population-based framework
Source: Endocr Connect. 2026 Aug 17;15(8):e260366. doi: 10.1530/EC-26-0366 (PMC13539411; doi:10.1530/EC-26-0366)
Supplement: Supplementary file 1 [file EC-26-0366_supplementary_figure_1.pdf]

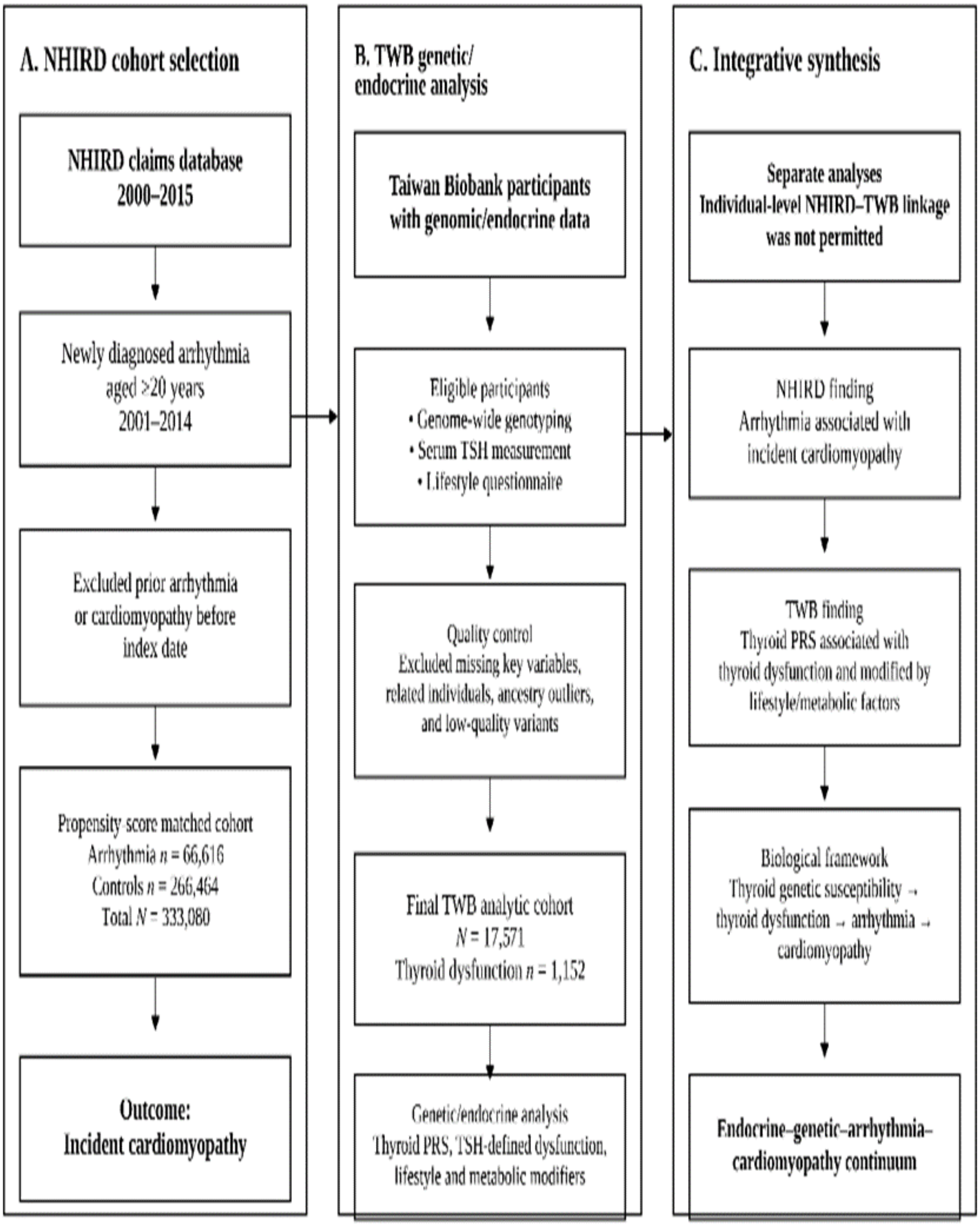

Figure S1. Flowchart of the integrative population-based study design
