# Supplementary material for: Arrhythmia and cardiomyopathy risk in Taiwan with complementary biobank evidence on thyroid genetic susceptibility: an integrative population-based framework
Source: Endocr Connect. 2026 Aug 17;15(8):e260366. doi: 10.1530/EC-26-0366 (PMC13539411; doi:10.1530/EC-26-0366)
Supplement: Supplementary file 2 [file EC-26-0366_supplementary_table_1.pdf]

**Table S1. Genome-wide significant loci associated with serum TSH and other thyroid-related traits in Taiwan Biobank participants**

| **Category** | **Chr** | **Lead SNP** | **Nearest gene / locus** | **Effect allele** | **$\beta$ for TSH** | **SE** | ***p*-value** | **Previously reported trait** |
|---|---|---|---|---|---|---|---|---|
| TSH-associated locus | 9 | rs1588635 | *PTCSC2 (FOXE1 locus)* | A | -0.0565 | 0.0051 | 5.86e-28 | Serum TSH |
| TSH-associated locus | 16 | rs4575545 | *MAFTRR (MAF)* | A | -0.0360 | 0.0034 | 1.47e-25 | Serum TSH |
| TSH-associated locus | 1 | rs10799824 | LOC105376819 (lncRNA) | A | -0.0471 | 0.0048 | 1.43e-22 | Serum TSH |
| TSH-associated locus | 1 | rs4915077 | *VAV3* | C | 0.0333 | 0.0036 | 2.00e-20 | Serum TSH |
| TSH-associated locus | 14 | rs189261858 | *TSHR (missense)* | A | 0.219 | 0.0262 | 7.42e-17 | Serum TSH |
| TSH-associated locus | 6 | Affx-28657309 | *VEGFA region* | G | -0.0300 | 0.0038 | 1.84e-15 | Serum TSH |
| TSH-associated locus | 2 | rs1861628 | *IGFBP-AS1* | A | -0.0248 | 0.0031 | 1.85e-15 | Serum TSH |
| TSH-associated locus | 5 | rs1382879 | *PDE8B* | T | -0.0301 | 0.0042 | 7.37e-13 | Serum TSH |
| TSH-associated locus | 6 | rs2983496 | *PDE10A* | A | -0.0456 | 0.0064 | 8.99e-13 | Serum TSH |
| TSH-associated locus | 14 | rs12101261 | *TSHR* | C | 0.0226 | 0.0032 | 1.84e-12 | Serum TSH |
| TSH-associated locus | 3 | rs4578973 | Intergenic | T | -0.0229 | 0.0033 | 6.83e-12 | Serum TSH |
| TSH-associated locus | 5 | rs2928167 | *PDE8B* | A | 0.0261 | 0.0040 | 4.54e-11 | Serum TSH |
| TSH-associated locus | 15 | rs3214696 | *DET1* | T | -0.0229 | 0.0036 | 3.56e-10 | Serum TSH |
| TSH-associated locus | 19 | rs4804416 | *INSR* | G | 0.0189 | 0.0032 | 2.88e-09 | Serum TSH |
| TSH-associ | 2 | rs1861630 | *IGFBP-AS* | T | -0.02 | 0.00 | 2.19e- | Serum |

| | | | | | | | | |
|---|---|---|---|---|---|---|---|---|
| ated locus | | | *1* | | 27 | 41 | 08 | TSH |

**Note:** This table summarizes the independent genome-wide significant lead SNPs ($p < 5\times10^{-8}$) associated with serum thyrotropin (TSH) levels in Taiwan Biobank participants. Independent signals were defined by LD clumping; nearby signals within the same locus were consolidated to a single lead SNP.
Genomic coordinates are reported on GRCh38. The nearest gene for each lead SNP was assigned by positional annotation on GRCh38. $\beta$ and standard error (SE) are reported on the analysis scale of the TSH phenotype. The effect allele corresponds to the tested allele (A1), and β is oriented to this allele.
Loci are classified into two categories: (1) TSH-associated loci, primarily associated with serum TSH regulation; and (2) Other thyroid-related loci, previously implicated in hypothyroidism, thyroid dysfunction, or broader thyroid hormone regulation. The previously reported trait is based on prior genome-wide association studies.
